# Resection for pancreatic cancer metastases contributes to survival: A case report with sequential tumor genotype profiling during the long-term postoperative course

**DOI:** 10.1097/MD.0000000000020564

**Published:** 2020-06-19

**Authors:** Hiroki Sato, Junpei Sasajima, Tetsuhiro Okada, Akihiro Hayashi, Hidemasa Kawabata, Takuma Goto, Kazuya Koizumi, Nobue Tamamura, Hiroki Tanabe, Mikihiro Fujiya, Shin-ichi Chiba, Mishie Tanino, Yusuke Ono, Yusuke Mizukami, Toshikatsu Okumura

**Affiliations:** a Department of Medicine; b Center for Advanced Research and Education; c Department of Surgical Pathology, Asahikawa Medical University, Asahikawa; d Institute of Biomedical Research, Sapporo Higashi Tokushukai Hospital, Sapporo; e Present address: Gastroenterology Medicine Center, Shonan Kamakura General Hospital, Kanagawa, Japan.

**Keywords:** metastasectomy, metastatic pancreatic cancer, pancreatic ductal carcinoma, post-operative survival, tumor genotyping

## Abstract

**Introduction:**

Surgical management is not a standard treatment option for metastatic recurrence of pancreatic adenocarcinoma. However, the surgical management of a solitary metastasis is useful in selected cases.

**Patient concerns:**

A 42-year-old woman was referred to our hospital on account of epigastric pain associated with a mass in the pancreatic body. The patient had a family history of branch duct-type intraductal papillary mucinous neoplasm of the pancreas.

**Diagnosis:**

The patient was diagnosed with pancreatic ductal adenocarcinoma (PDA) complicated with pancreatitis due to pancreatic duct involvement.

**Interventions:**

The patient underwent distal pancreatectomy, and pathological examination revealed a tubular adenocarcinoma. Solitary liver and lung metastatic tumors were found 6 and 43 months after the initial presentation, respectively, and sequential metastasectomies were performed.

**Outcomes:**

The patient survived until 8 years after her initial presentation. The genetic profiles of the resected specimens, primary PDA, and recurrent tumors in the liver and lung possessed identical KRAS mutations at codon 12, whereas there were no mutations in the main tumor suppressor genes, such as TP53, CDKN2A, and SMAD4. Multiplex polymerase chain reaction-based microsatellite instability assay demonstrated microsatellite stability.

**Conclusion:**

In our case, the patient with pancreatic adenocarcinoma survived for over 8 years following the resection of the primary tumor and resections of metachronous metastatic tumors. The outcome of PDA may be associated with the genetic profile that regulates its biological behavior. Operative management of solitary metastatic tumors may be a therapeutic options for selected patients with pancreatic cancer.

## Abstract 2

Supplemental Digital Content is available in the text

## 1 Introduction

Pancreatic ductal adenocarcinoma (PDA) is a lethal disease that often relapses even after curative resection is performed. Although adjuvant chemotherapy is recommended, the median relapse-free survival duration was <2 years, and recurrence was observed in about two-thirds of patients within 5 years after surgery.^[1,2]^ Metastasis is a systemic disease in general, and surgical intervention for metastatic recurrence is not a reasonable therapeutic option in most cases. However, previous reports demonstrated the survival benefits of the surgical resection of solitary metastases of PDA.^[3,4]^

A particular ubiquitous mutation in *KRAS* acts as an initiating genetic event during pancreatic carcinogenesis, and subsequent loss-of-function of tumor suppressor genes such as *TP53*, *CDKN2A,* and *SMAD4* leads to the progression of the precursors of invasive and metastatic disease.^[5]^ A previous study showed that the number of these mutations significantly correlated with the postoperative survival of patients with PDA.^[6]^ Recent genetic studies also identified considerable diversity in mutation profiles among PDAs, which may correspond broadly to molecular subtypes.^[7–10]^ Clarity regarding the clinical significance of the genetic landscape of PDA will be necessary to predict potent long-term survival and to select patients for surgical interventions even when synchronous or metachronous metastasis is evident.

Here, we describe the case of a patient with PDA, who has survived for >8 years after resection of the primary tumor followed by subsequent surgical interventions for solitary recurrent tumors in the liver and lung. We further describe how long-term survival was achieved with sequential resections of both primary and recurrent tumors in the liver and lung, which showed identical *KRAS* mutations with no other mutations identified among the main tumor suppressor genes. The unusual and favorable clinical course of the patient may be associated with the rare genetic profiling of cancer cells.

## 2 Case presentation

**Figure 1:**
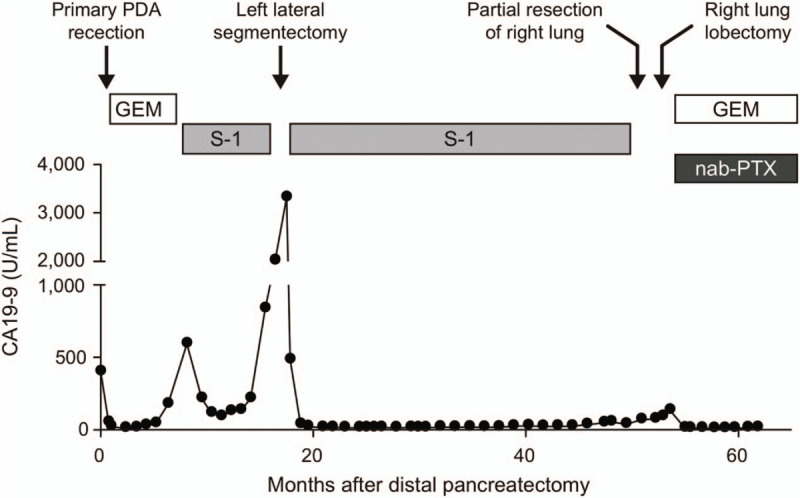
Timing of surgical interventions during the clinical course of the patient. Fourteen months after distal pancreatectomy, left lateral segmentectomy was performed. Additional lung metastasis was also resected 43 months after the initial presentation. Serum CA19-9 levels and chemotherapeutic agents used are shown.

**Figure 2:**
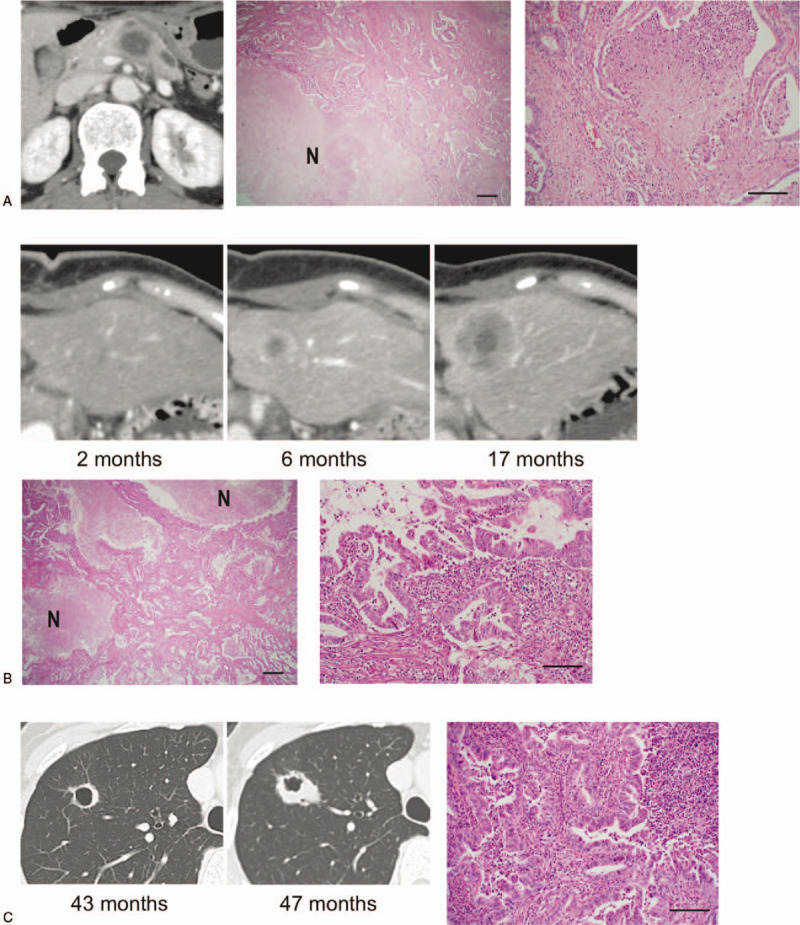
Imaging and histological findings of the primary and metastatic tumors. Computed tomography scan and histological findings of the primary tumor (A), liver metastasis (B), and right lung (C). N: large necrotic areas at the center of the tumor. Scale bars; 500 μm (left) and 100 μm (right panel).

A 42-year-old woman was referred to our hospital on account of epigastric pain. Her mother had been diagnosed with a branch-duct type intraductal papillary mucinous neoplasm of the pancreas at the age of 59 years, with no evidence of malignancy. A blood test showed an elevated carbohydrate antigen 19–9 (CA19-9) level (Fig. 1). A computed tomography (CT) scan revealed fluid collection in front of a low-density mass of the pancreatic body with a diameter of 3 cm (Fig. 2A). The tumor lacked enhancement in the majority of the central aspect, consistent with central necrosis. The patient was diagnosed with PDA complicated with pancreatitis due to pancreatic duct involvement. The CT scan and magnetic resonance imaging detected no distant metastasis, and the patient underwent distal pancreatectomy. Pathological examination of the resected specimen revealed moderately differentiated tubular adenocarcinoma with extensive necrosis (Fig. 2A), staged as pT3 N0 M0 (Stage IIA), and the surgical margin was negative for malignancy.

The level of CA19-9 normalized postoperatively, and adjuvant chemotherapy with gemcitabine was initiated. However, the levels were reelevated at 6 months after distal pancreatectomy (Fig. 1). A CT scan revealed a solitary low-density mass in the left lateral segment of the liver with massive necrosis, which was a phenocopy of the primary pancreatic tumor (Fig. 2B). Due to the tumor recurrence in the liver, S-1 (tegafur/gimeracil/oteracil potassium) was administered as second-line therapy. The level of CA19-9 decreased after the chemotherapy but was found to be reelevated 14 months after the distal pancreatectomy (Fig. 1).

As the liver metastasis slowly enlarged without any radiographic evidence of additional distant metastases, surgical resection of the liver metastasis was performed. Pathological examination of the resected specimen again revealed moderately differentiated tubular adenocarcinoma with substantial necrosis, consistent with liver metastasis of PDA (Fig. 2B).

Following chemotherapy with S-1, a solitary mass with a central cavity in the right pulmonary lobe was detected at 43 months after the initial presentation (Figs. 1 and 2C). The patient underwent thoracoscopic partial resection for pathological evaluation; PDA was histologically confirmed in the resected lung specimen (Fig. 2C). The patient experienced local pulmonary recurrence 2 months after the resection (Fig. 1). Upper lobectomy of the right lung was performed, followed by chemotherapy with nab-paclitaxel plus gemcitabine. Although local relapse was detected after lobectomy of the lung, the patient is still alive 8 years after her initial presentation.

To assess the genetic events that occurred during the long-term course after resection of the primary PDA, we characterized the primary tumor and liver and lung metastases by targeted sequencing based on polymerase chain reaction (PCR) amplicons using genomic DNA extracted from formalin-fixed paraffin embedded (FFPE) specimens, discussed as follows.

### 2.1 Samples and DNA extraction

Genomic DNA was extracted from FFPE sections of nontumor tissue (spleen), primary PDA, and liver/lung metastases using the GeneRead DNA FFPE kit (Qiagen, Hilden, Germany). The quantity of isolated genomic DNA was assessed using the Qubit 2.0 Fluorometer (Thermo Fisher Scientific, Waltham, MA).

### 2.2 Targeted amplicon sequencing

The mutation profiles of primary and liver/lung metastatic tumors were determined via targeted sequencing based on PCR amplicons as described previously.^[11,12]^ Ten to 60 nanogram of genomic DNA was amplified via PCR using Ion AmpliSeq Cancer Hotspot Panel v2 and a custom designed panel (both obtained from Thermo Fisher Scientific). Ion Ampliseq Cancer Hotspot Panel v2 is designed to amplify 207 amplicons covering major hotspots of 50 oncogenes. The custom designed amplicons cover 18 genes frequently mutated in PDA (*ARID1A*, *BRAF*, *CDKN2A*, *CTNNB1*, *GNAS*, *IDH1*, *KDM6A*, *KRAS*, *MAP2K4*, *PIK3CA*, *RBM10*, *SF3B1*, *SMAD4*, *STK11*, *TGFBR1*, *TGFBR2*, *TP53*, and *RNF43*) (see details in the Supplementary Table), containing 440 DNA oligonucleotide primers designed for 220 amplicons.^[12]^

Sequencing was performed using an Ion Personal Genome Machine System and the Ion Personal Genome Machine Sequencing 200 Kit (both obtained from Thermo Fisher Scientific) according to the manufacturer's instructions. Sequence reads were demultiplexed, quality-filtered, and aligned to the human reference genome (Genome Reference Consortium Human Build 37) using the Torrent Suite software (version 5.0.4; Thermo Fisher Scientific). Variants were identified using the Variant Caller software (version 5.0.4.0; Thermo Fisher Scientific), and alignments were visually checked with the Integrative Genomics Viewer software (version 2.3.59; Broad Institute, MA).

### 2.3 Microsatellite instability analysis

The microsatellite phenotype of each tumor was assessed using the Promega microsatellite instability (MSI) Analysis System (version 1.2; Promega Madison, WI). This system uses 5 mononucleotide markers to identify MSI in tumor and normal tissue DNA (BAT-25, BAT-26, NR-21, NR-24, and MONO-27), and 2 pentanucleotide markers (Penta C and Penta D) to identify whether the tumor and normal DNA specimens were obtained from the same patient. A Compact CE Sequencer (DS3000; Hitachi High-Tech Corp., Tokyo, Japan) was used to separate the products obtained from fluorescently labeled PCR, and the data were analyzed using GeneMaker 3.0 software (SoftGenetics, State College, PA). Tumors were classified as MSI-high if they showed instability in at least 2 of 5 markers, MSI-low if instability was observed in 1 of 5 markers, and microsatellite stable if no instability was detected in the 5 markers.^[13]^

All 3 tumors possessed identical *KRAS* mutations at codon 12 (p.G12V, COSM520), and the liver and lung tumors were genetically identical to the primary PDA. Interestingly, no mutation was found in the main tumor suppressors of pancreatic tumorigenesis (eg, *TP53*, *SMAD4*, and *CDKN2A*) and other genes commonly mutated in PDA. Also, new mutations were not demonstrated to have sequentially accumulated in the liver and lung metastases. Both primary and recurrent tumors were microsatellite stable on multiplex PCR-based MSI assay (Supplementary Fig. 1).

### 2.4 Ethics statement

The protocol of the present study was approved by the institutional review board of Asahikawa Medical University (IRB approval no. 15002). Written informed consent was obtained from the patient before the genetic study for publication.

## 3 Discussion

Here, we described the case of a patient with PDA, who has survived for >8 years after sequential resection of the primary tumor and solitary metastatic tumors. Since metastatic relapse of cancer is commonly considered as a systemic disease, surgical intervention for the recurrent tumor is not generally offered, and the patients usually receive chemotherapy. However, recent studies showed that metastasectomy improves long-term survival and even provides a chance of cure in some types of cancer.^[14–21]^ Based on these observations, surgical resection has been recognized as a therapeutic option for metastatic lesions.

Studies on the surgical resection of solitary metastatic lesions of PDA have been conducted on a small number of patients, and the preliminary results indicate a survival benefit.^[3,4]^ In pulmonary metastasis, Arnaoutakis et al^[3]^ reported that median cumulative survival was significantly improved with pulmonary metastasectomy, which is 51 months, compared to 23 months in the nonpulmonary resected group. This study showed successful outcomes for recurrence of pulmonary metastasis of PDA and the 3 proposed 3 indication criteria were as follows: a relatively long interval between the initial surgery and relapse, isolated and stable disease over time, and favorable response to systemic therapy. Shrikhande et al reported that when the patients with liver metastasis received synchronous liver resection, the median overall survival was 11.4 months. The other case-control study showed that for hepatic/lung oligometastatic patients with PDA, the median overall survival in patients who underwent metastasectomy was 2.7 years, where as in patients who received nonmetastasectomy treatment was 0.98 years.^[22]^ In the present case, solitary liver metastasis and pulmonary metastatic relapse were discovered 6 months and 43 months after the initial resection, respectively, and systemic chemotherapy controlled both metastatic tumors well. Thus, synchronous or metachronous resection of pulmonary/liver metastases may help into improving the patients’ survival if they are in stable disease with conventional chemotherapy.^[23]^

Metastatic PDA lesions that are successfully controlled via surgery may belong to a subset that is more favorable at the molecular level.^[24,25]^ Therefore, we took a genetic approach to identify the mutation profile. Identical *KRAS* mutations were found in all 3 lesions, suggesting that the metastasis likely originated in the primary PDA. It is generally assumed that tumor cells acquire additional mutations during progression. However, other mutations commonly found in PDAs such as *TP53*, *CDKN2A*, and *SMAD4* were neither demonstrated in the primary PDA nor in the metastases. In addition, the mutation burden did not increase during the course, although a limited number of genes were analyzed in the current case. The prevalence of MSI and its association with the outcomes of pancreatic cancer may vary depending on the method used.^[26]^ Due to the relatively favorable prognosis of MSI-high PDA, particularly among patients with associated Lynch syndrome, the MSI phenotype determined in the present case resulted in microsatellite stability.

Considering the early onset of PDA in this patient, genetic susceptibility that by-passed the common tumor suppressor pathways of PDA may have played a role in tumor development and metastatic progression. Recent studies showed the significance of germline cancer susceptibility variants and, somatic second hits, during pancreatic carcinogenesis. A subset of PDA patients with such genetic susceptibility may have vulnerability not only against cancer immune therapy but also multiple resections. The significance of this case report is to uncover mutation profiles in both primary and metastatic lesions, and further germline testing should be considered.^[27–29]^

Accumulation of a large number of cases will be required to analyzed to identify factors crucial in determining their outcome and to achieve optimal treatment.^[30]^

This report has several limitations. First, since we did not perform a comprehensive molecular analysis of the primary and metachronous tumors, it is unclear whether the unique mutation signature may be related to the favorable outcome of the patient. Second, it is unclear how the sequential chemotherapy followed by metastasectomies affected the patient's survival and tumor genome.

## 4 Conclusions

We described a case of pancreatic adenocarcinoma that survived for >8 years following resection of the primary tumor and metachronous metastatic tumors in the liver and lung. The outcomes of PDA may be associated with a genetic profile that regulates its biological behavior. Operative management of solitary metastatic tumors may be considered as a therapeutic option for selected patients with pancreatic cancer.

## Acknowledgments

The authors to thank Munehiko Ogata (Sapporo Higashi Tokushukai Hospital) for preparing the tissue samples and performing the genetic analysis. We are also grateful to Dr. Hidenori Karasaki (Sapporo Higashi Tokushukai Hospital) for the helpful discussions.

## Author contributions

**Data curation:** Shin-ichi Chiba.

**Investigation:** Hiroki Sato, Junpei Sasajima, Tetsuhiro Okada, Akihiro Hayashi, Hidemasa Kawabata, Takuma Goto, Nobue Tamamura, Hiroki Tanabe, Shin-ichi Chiba, Mishie Tanino, Yusuke Ono, Yusuke Mizukami.

**Project administration:** Hiroki Sato, Takuma Goto, Hiroki Tanabe, Yusuke Mizukami.

**Supervision:** Junpei Sasajima, Hiroki Tanabe, Mikihiro Fujiya, Shin-ichi Chiba, Mishie Tanino, Yusuke Ono, Toshikatsu Okumura.

**Validation:** Hiroki Tanabe, Mishie Tanino.

**Writing – review & editing:** Hiroki Sato, Mikihiro Fujiya, Yusuke Mizukami.

## Supplementary Material

Supplemental Digital Content

## Supplementary Material

Supplemental Digital Content
