# Supplementary material for: Resection for pancreatic cancer metastases contributes to survival: A case report with sequential tumor genotype profiling during the long-term postoperative course
Source: Medicine (Baltimore). 2020 Jun 19;99(25):e20564. doi: 10.1097/MD.0000000000020564 (PMC7310851; doi:10.1097/MD.0000000000020564)
Supplement: Supplemental Digital Content [file medi-99-e20564-s001.docx]

**Supplementary Table 1: Targeted regions of the 18 genes explored in the AmpliSeq custom panel**

| Gene | No. of amplicons | Total amplicon length (bp) |
| --- | --- | --- |
| *KRAS* | 4 | 309 |
| *TP53* | 14 | 1,317 |
| *CDKN2A* | 3 | 307 |
| *SMAD4* | 11 | 912 |
| *GNAS* | 2 | 170 |
| *RNF43* | 36 | 3,349 |
| *BRAF* | 4 | 342 |
| *PIK3CA* | 4 | 311 |
| *STK11* | 6 | 553 |
| *IDH1* | 2 | 153 |
| *CTNNB1* | 2 | 152 |
| *MAP2K4* | 12 | 978 |
| *TGFBR1* | 21 | 1,712 |
| *TGFBR2* | 12 | 1,071 |
| *ARID1A* | 47 | 2,934 |
| *SF3B1* | 8 | 628 |
| *RBM10* | 15 | 1,371 |
| *KDM6A* | 17 | 1,394 |
