# Supplementary figures and images for: Resection for pancreatic cancer metastases contributes to survival: A case report with sequential tumor genotype profiling during the long-term postoperative course
Source: Medicine (Baltimore). 2020 Jun 19;99(25):e20564. doi: 10.1097/MD.0000000000020564 (PMC7310851; doi:10.1097/MD.0000000000020564)

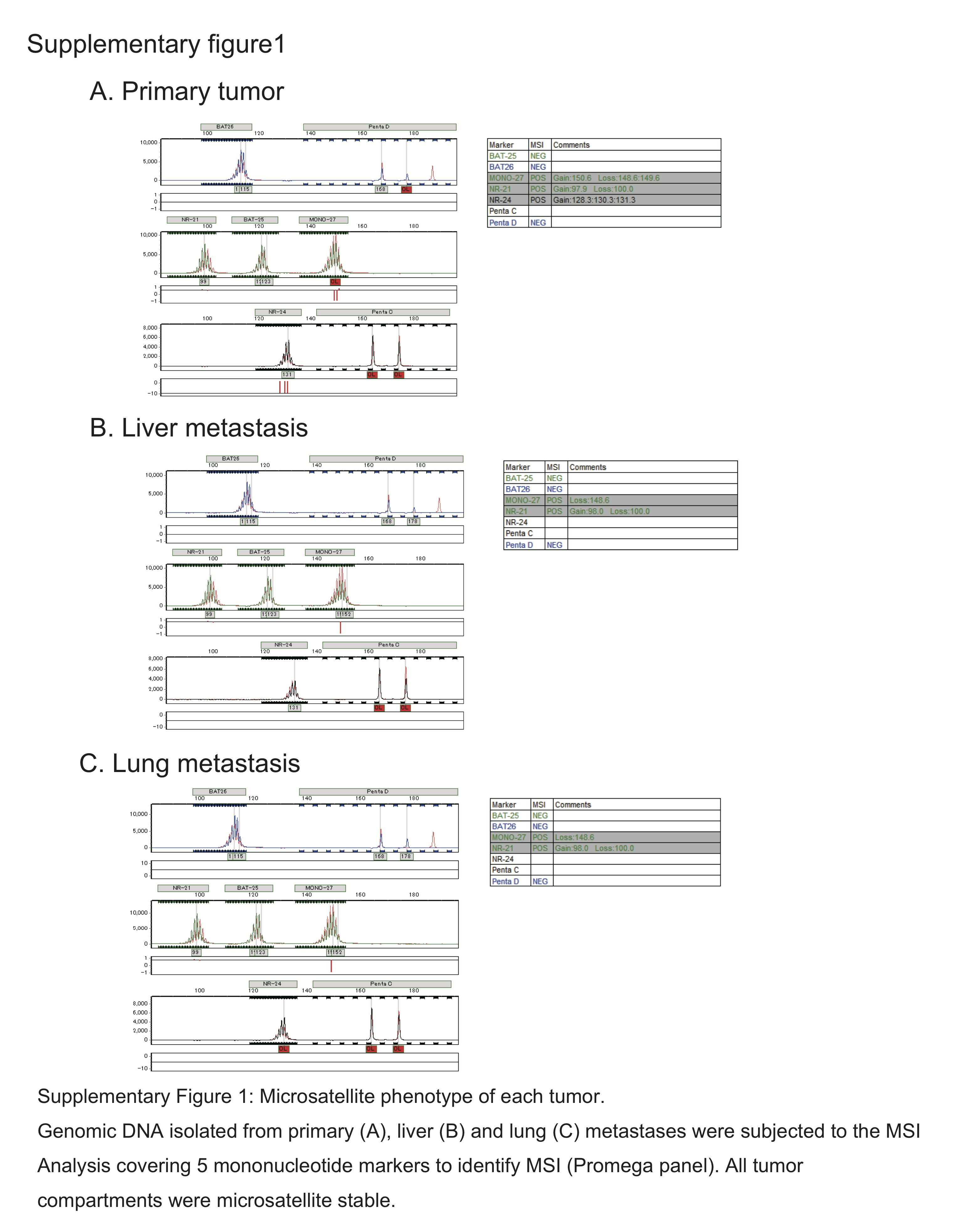

Supplement: Supplemental Digital Content [file medi-99-e20564-s002.tiff]
